# Remarkably enhanced photovoltaic effects and first-principles calculations in neodymium doped BiFeO_3_

**DOI:** 10.1038/srep45164

**Published:** 2017-03-24

**Authors:** Yi-Ting Peng, Shan-Haw Chiou, Ching-Hung Hsiao, Chuenhou (Hao) Ouyang, Chi-Shun Tu

**Affiliations:** 1 Department of Materials Science and Engineering, National Tsing Hua University, Hsinchu, Taiwan 300, Republic of China; 2 Material and Chemical Research Laboratories and Nanotechnology Research Center, Industrial Technology Research Institute, Hsinchu, Taiwan 310, Republic of China; 3 Department of Physics, Fu Jen Catholic University, New Taipei City, Taiwan 24205, Republic of China

## Abstract

Remarkably enhanced photovoltaic effects have been observed in the heterostructures of *p*-type A-site Nd^3+^-doped BiFeO_3_ (Bi_0.9375_Nd_0.0625_)FeO_3_ (or BFONd) polycrystalline ceramics and the n-type ITO thin film. The maximum power conversion is ~0.82%, which is larger than 0.015% in BiFeO_3_ (BFO) under blue-ultraviolet irradiation of wavelength λ = 405 nm. The current-voltage (I-V) characteristic curve suggests a p-n junction interface between the ITO thin film and BFO (or BFONd) ceramics. The band gaps calculated from first-principles for BFO and BFONd are respectively 2.25 eV and 2.23 eV, which are consistent with the experimental direct band gaps of 2.24 eV and 2.20 eV measured by optical transmission spectra. The reduction of the band gap in BFONd can be explained by the lower electronic Fermi level due to acceptor states revealed by first-principles calculations. The optical calculations show a larger absorption coefficient in BFONd than in BFO.

Multiferroic BiFeO_3_ (BFO) possesses a ferroelectric Curie temperature of T_c_ ~ 1103 K and a G-type antiferromagnetic order (Neel temperature T_N_ ~  643 K) with a spatially modulated spin structure. There is also the existence of weak Dzyaloshinskii-Moriya-type ferromagnetism^1^. Ferroelectrics have recently received lots of interest for potential applications in photovoltaic (PV) responses^2^. The major advantage of BFO is a relatively small band gap (2.2~2.8 eV)^3^,^4^,^5^,^6^,^7^,^8^,^9^ in the visible spectrum amongst the other ferroelectric oxides with large band gaps (>3 eV), including LiNbO_3_, BaTiO_3_, SrTiO_3_, and Pb(Zr,Ti)O_3_^3^,^10^. Several mechanisms of photovoltaic effects have been proposed for BFO thin films and crystals, including the bulk photovoltaic effect^11^, the domain-wall model^12^, and lastly, the semiconductor-like p-n junction model^4^, the one adopted in this study. Our recent studies suggested that domain structure and hybridization between the O 2*p* and Fe 3*d* orbitals play important roles for the PV responses in the ITO/(Bi_1−*x*_Sm_*x*_)FeO_3_ ceramic/Au heterostructure^13^.

BFO thin film has been reported as a p-type semiconductor, revealed by the rectifying current density versus voltage (J-V) behavior due to Bi deficiency during the high-temperature sintering process^14^. The first-principles calculation found that Bi vacancies have lower defect formation energy than oxygen vacancies, suggesting that Bi vacancies are the acceptor defects that cause BFO to become a p-type semiconductor^15^. However, BFO exhibited serious current leakage due to oxygen vacancies causing a valence shift of Fe^3+^ → Fe^2+^ 16 that formed shallow energy centers (defect states)^17^. This is a problem in the industrial application of photovoltaic devices. The density-functional-theory (DFT) calculation and X-ray absorption spectroscopy revealed that the A-site rare-earth lanthanum (La) doping in BFO can reduce the leakage current^18^. It has been reported that doping can be one of the most effective methods used to improve ferroelectric and magnetic properties^19^,^20^,^21^,^22^, as the Bi-site substitution can effectively control the volatility of Bi atoms with reduction of oxygen vacancies^23^ and enhancement of photovoltaic effects^24^,^25^,^26^. For photovoltaic applications, the BFO should be able to absorb as much light as possible to generate a photocurrent, which requires a lower band gap and large absorption coefficient^27^. The photocurrent density under irradiation with a certain wavelength can be described by an empirical Glass Law^28^:


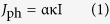


where α is the optical absorption coefficient, κ is a material-dependent Glass coefficient, and I is irradiation intensity. The photocurrent density J_ph_ is linearly proportional to the absorption coefficient. The relation between α and an optical band gap *E*_g_ can be estimated using the Tauc relation, i.e.^5^,^29^


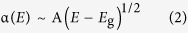


where A is a material-dependent constant and *E* is the incident photon energy. According to Eqs (1) and (2), one can extrapolate a larger photocurrent density by increasing the absorption coefficient with a small band gap.

In the study, enhanced photovoltaic effects have been observed in the ITO/BFONd ceramic/Au heterostructure under blue-ultraviolet irradiation of λ = 405 nm, where the power conversion efficiency reached ~0.82%, higher than that of most BFO materials^13^,^30^,^31^,^32^,^33^,^34^. It opens promising future applications for optical devices such as photo-driven actuators and optical sensors. The first-principles calculation suggests that the reduction of the band gap resulted from a lower electronic Fermi level responsible for the enhanced photovoltaic responses. The calculated band gaps of 2.25 eV (for BFO) and 2.23 eV (for BFONd) are thus consistent with experimental values of ~2.24 eV and ~2.20 eV, respectively.

## Experimental

(Bi_0.9375_Nd_0.0625_)FeO_3_ (BFONd) ceramics were prepared using the solid state reaction method, in which Bi_2_O_3_, Nd_2_O_3_, and Fe_2_O_3_ powders (purity ≥ 99.0%) were weighed in a ratio of 0.95:0.05:1.0. The powders were mixed in an agate mortar for more than 24 hours, with alcohol as a medium. The dried mixtures were calcined at 800 °C for 3 hours and then sintered at 870 °C for three hours. X-ray diffraction (XRD) spectra were performed using a Shimadzu-XRD6000 X-ray diffractometer. The Rietveld-refinement method, used in the General Structure Analysis System (GSAS), was performed for crystallographic analysis and the determination of Nd concentration^35^. Scanning transmission electron microscopy (STEM) imaging was performed using Cs corrected-field-emission TEM (Ultra-HRTEM). For photovoltaic (PV) measurements, ITO (~100 nm) and Au thin films were deposited on the ceramic surfaces by dc sputtering as top and bottom electrodes respectively. The irradiated area (also known as the electrodes area) of the samples is about 0.15 cm^2^. A diode laser (*λ* = 405 nm) was used as the illumination source, and the laser beam was incident perpendicular to the sample surface with the ITO film.

## Calculations

Ab initio calculations were performed using the accurate full-potential projector augmented wave (PAW) method^36^ as implemented in the Vienna *ab initio* simulation package (VASP)^37^,^38^. Our first-principles approach is based on the LSDA+U method^39^,^40^ with a Hubbard parameter from 5.0 eV to 9.0 eV and an effective on-site exchange interaction of J = 0.9 eV for Fe 3*d* electrons^41^. A large plane-wave cutoff of 500 eV^41^ was used throughout the calculation. Brillouin zone integrations were performed with the tetrahedron method, using Blöchl corrections in a 3 × 3 × 3 Monkhorst–Pack k-point mesh centered at Γ^42^ and the k-points mesh was 10 × 10 × 10 for density of states (DOS) calculations. The unit cell of rhombohedral *R*3*c* space group for BiFeO_3_ was extended to a 2 × 2 × 2 super ell containing 80 atoms which includes 16 bismuth atoms, 16 iron atoms and 48 oxygen atoms to simulate the Nd doping atomic ratio. All structures were full relaxed. The formation energy calculation was performed with one Nd atom substitutes doping the A(Bi)-site, or Fe-site, or interstice B, C or D position to simulate. Thus, the Nd concentration is 1.25% atomic ratio for a 2 × 2 × 2 super cell model. The DOS of BiFeO_3_ and 1.25% atomic ratio Nd doped BiFeO_3_ were calculated also. The optical absorption coefficient was obtained by using the equation 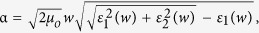
43 which can be used in a nonconducting dispersive medium. Here *w* is photon frequency, *μ*_o_ is the permeability of free space, *ε*_1_ and *ε*_2_ are frequency-dependent real and imaginary parts of dielectric permittivity from the VASP output of the first-principle calculation.

## Results and Discussion

**Figure 1:**
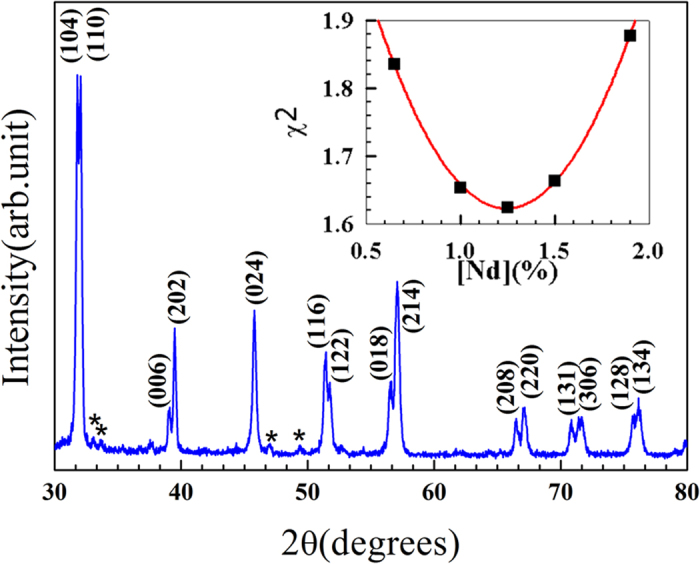
Powder XRD spectra of BFONd. The inset is the plot of Nd concentration vs. χ^2^ with a fitting curve (red line) and the minimum χ^2^ occurs at Nd concentration ~1.25%.

**Figure 2:**
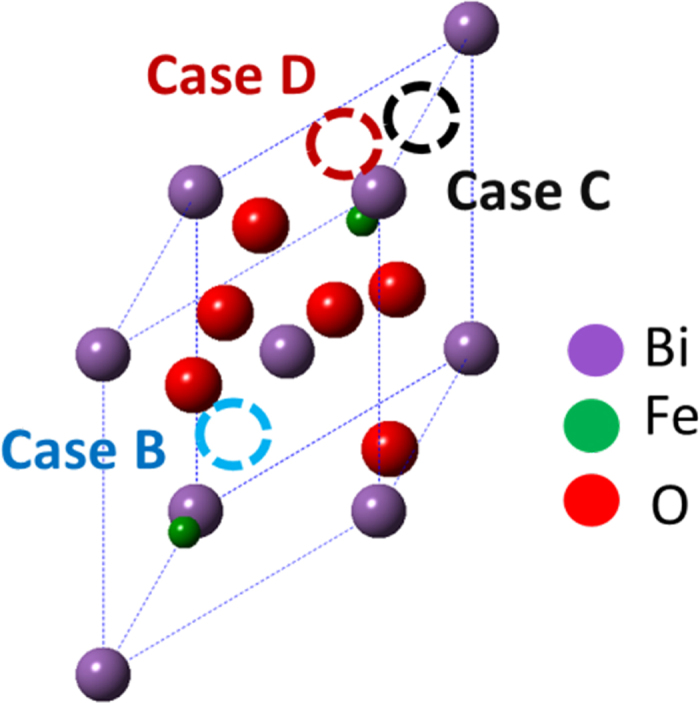
Coordinate interstitial sites of B, C, and D: (0.360385, 0.3608385, 0.360385), (0.851385, 0.851385, 0.851385), and (0.8006925, 0.8006925, 0.9256925).

The optical band gap depends strongly on the proper choice of the Hubbard parameter U for Fe^41^. It was found that when U = 7.5 eV, there was the smallest deviation between experimental and calculated band gaps with J = 0.9 eV as shown in Table S1. Figure 1 shows the powder XRD spectra of BFONd and Nd concentration vs. χ^2^ (goodness of fit) from the Rietveld-refinement fitting curve (red line). The exsitence of a second phase of Bi_2_Fe_4_O_9_ resulted in a Nd-rich situation, as was expected initally. In order to find the Nd concerntration, we uesd the Rietveld refinement to calculate the least squared χ^2^ as a function of Nd concentration [Nd]. The calculated Nd concerntration [Nd] corresponding to the minimum χ^2^ occurrs at ~1.25% (Fig. 1). The fractional coordinates and occupancies of the Bi, Fe, O, and Nd atoms at the minimum, χ^2^~1.625, are given in Table 1. It is important to know that Nd atom may either substitute the Bi-site or appear at the interstitial sites, as illustrated in Fig. 2. Three interstitial-site cases (B, C, and D) have been considered. The Rietveld refinements indicate negative occupancies in these Nd interstitial sites, as given in Table S2, suggesting that Nd substitution does not occupy interstitial sites.

**Figure 3:**
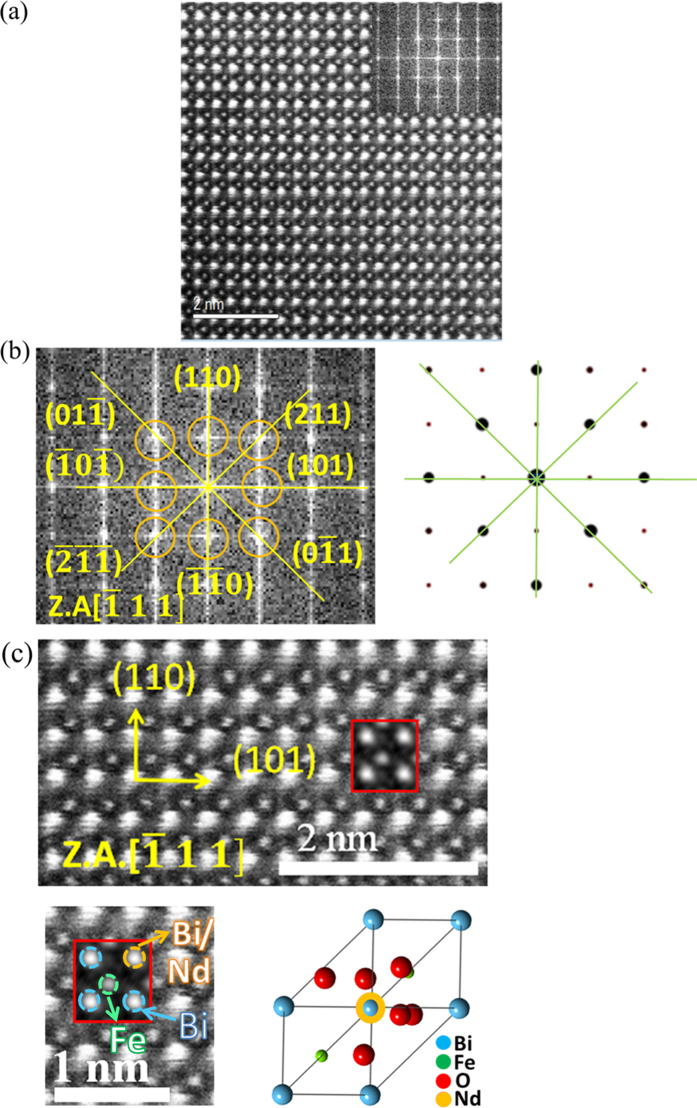
(**a**) Dark-field STEM image and diffraction pattern by fast Fourier transform for BFONd, (**b**) diffraction pattern and simulation along the 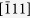 zone axis, (**c**) dark-field STEM image simulation consistent with experiment image along the 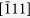 zone axis.axis.

The dark-field STEM images and simulation along the 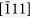 zone axis are given in Fig. 3. The angles of the simulated diffraction pattern are the same as the experimental diffraction pattern, and d spacings are in the 5% deviation range (Fig. 3(b)). The STEM image simulation in Fig. 3(c) agrees with the actual STEM image, revealing a rhombohedral R3c space group with lattice parameters of a = b = c = 5.63447 Å and α = 59.3498°, as well as an Nd substitution of an A-site Bi atom^44^. From the relaxation of first-principles calculation as shown in Table 2, the total free energy of A(Bi)-site Nd substitution with 1.25% [Nd] in 80 atoms has the smallest energy. This indicated a more thermodynamic and stable structure compared with the substituted-Fe site or interstitial-site cases and with lattice parameters of 5.6843 Å (super cell 11.3686 Å) and θ = 59.243°. These results are consistent with the Rietveld-refinement analysis and the STEM image.

**Figure 4:**
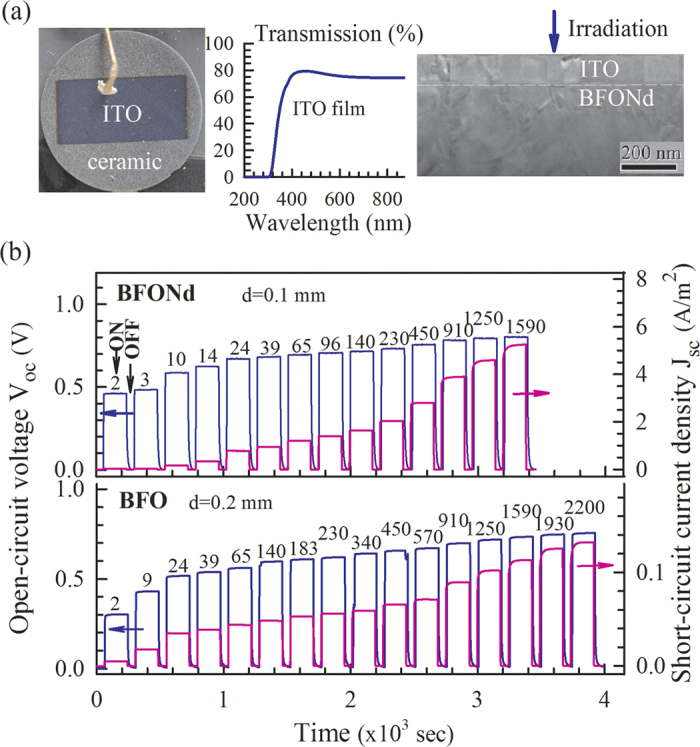
(**a**) Ceramic disk with ITO electrode, optical transmission of ITO film, and bright-field TEM image near the interface between ITO film and ceramic. (**b**) Open-circuit voltage V_oc_ and short-circuit current density J_sc_ as light was switched on and off with increasing irradiation intensity (in unit of W/m^2^) labeled on the tops of illuminations.

**Figure 5:**
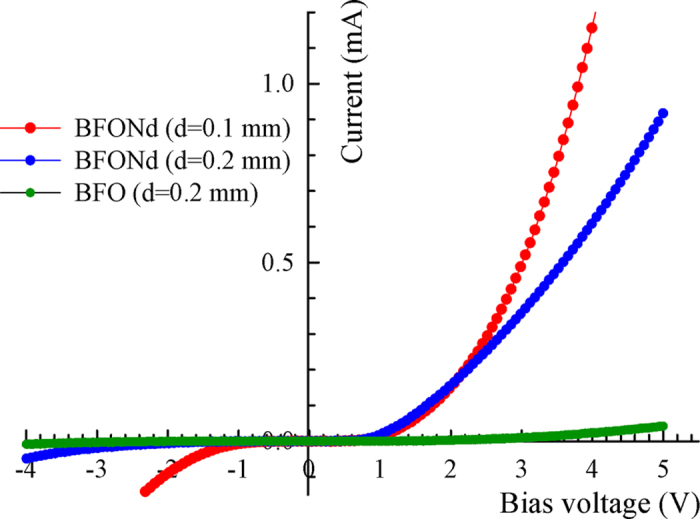
Characteristic curve of current vs. voltage (I-V) without irradiation.

**Figure 6:**
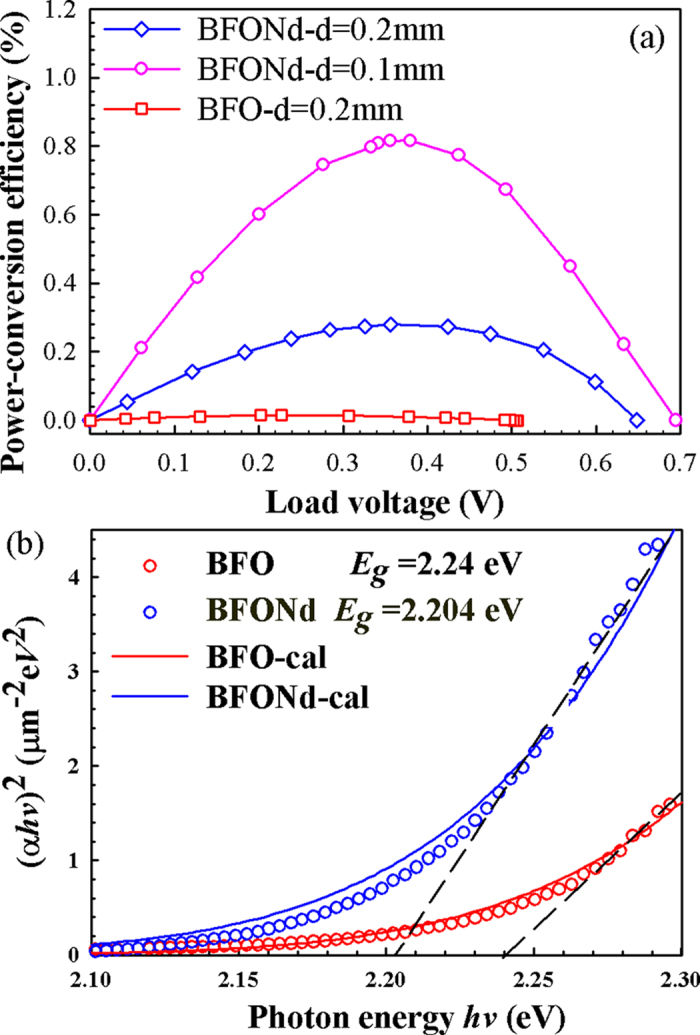
(**a**) Power conversion efficiency (η) of BFONd (pink line with thickness d = 0.1 mm and blue line with d = 0.2 mm) and BFO (red line with d = 0.2 mm). The maximal η values for BFO (d = 0.2 mm), BFONd (d = 0.1 mm) and BFONd (d = 0.2 mm) are respectively 0.015%, 0.816%, and 0.279%. (**b**) (αhν)^2^ vs. hν (photon energy) for BFO (red circles) and BFONd (blue circles), where solid lines are the calculation result.

Figure 4(a) shows the ceramic disk with ITO electrodes used for photovoltaic (PV) measurements, the wavelength-dependent optical transmission of the ITO thin film, and the bright-field TEM image near the interface region between ITO film and the BFONd ceramics. The optical transmission of the ITO film is about 80% at an irradiation of λ = 405 nm. Figure 4(b) illustrates the open-circuit voltage (V_oc_) and the short-circuit current density (J_sc_) as irradiation was sequentially switched on and off with increasing irradiation intensity (I) for BFO (thickness d = 0.2 mm) and BFONd (thickness d = 0.1 mm). The characteristic curves of the current vs. the bias voltage were measured without irradiation (Fig. 5), suggesting a p-n-junction-like behavior between the ITO thin film and BFO (or BFONd) ceramics. As shown in Fig. 6(a), the power-conversion efficiency (η = *P*_out_/*P*_in_) of BFONd with an optimized thickness d = 0.1 mm can reach ~0.82%, the highest among major ferroelectric and BFO materials with varying amounts of electrodes^13^,^30^,^31^,^32^. For sample thickness of d = 0.2 mm, the η in BFONd is 19 times higher than that of BFO. A detailed comparison of photovoltaic studies in major perovskite ferroelectrics and p-type BFO materials can be found in our previous study^13^. In this work, the ITO/BFONd/Au heterostructure has demonstrated comparable photovoltaic effects, including open-circuit voltage (V_oc_), short-circuit current density (Jsc), and power-conversion efficiency (η).

Figure S1 shows the optical transmission spectra of BFO and BFONd as a function of irradiation wavelength. The curves of (αhν)^2^ vs. hν (photon energy) for BFO (red circles) and BFONd (blue circles) are plotted in Fig. 6(b), where circles represent experimental data and solid lines represent the calculated results. As indicated by the dashed lines in Fig. 6(b), the experimental band gaps of BFO and BFONd are respectively about 2.24 eV and 2.20 eV. The first-principles density functional calculation suggested that oxygen vacancies in BFO can shift the optical absorption to lower energy^45^,^46^. The different band gaps between this study and earlier reports^3^,^4^,^5^,^6^,^7^,^8^ may be associated with oxygen vacancies.

To explain the enhanced photovoltaic effects in the ITO/BFONd/Au heterostructure, the band structures, DOS, and projection density of states (PDOS) of BFO and BFONd in the rhombohedral *R*3*c* structure are given in Fig. S2, indicating direct band gaps of 2.25 eV and 2.23 eV for BFO and BFONd, respectively. The computed Brillouin zone path is Γ(0,0,0); F (1/2, 1/2, 0); L (1/2, 0, 0); Z (1/2, 1/2, 1/2) for the rhombohedral *R*3*c* structure^47^.

The calculated band gaps with U = 7.5 eV are consistent with the measured values. The A site Nd doping causes a reduction of the band gap from *E*_g_~2.25 eV in BFO to *E*_g_~2.23 eV in BFONd. From the density of states for BFO in Fig. S2(a), the spin-up and spin-down configurations exhibit symmetric distributions, confirming an antiferromagnetic tendency as suggested by the linear hysteresis curve of magnetization vs. the magnetic field in BFO^48^. As evidenced in Fig. S2(b), the DOS of BFONd exhibits an asymmetric configuration, which may be the reason for enhanced magnetic properties in some doped BFO materials^21^,^22^.

According to Eqs (1) and (2) for the smaller band gap *E*_g_ in BFONd, a larger α and photocurrent density can be expected in BFONd when compared with BFO. The correlation of the open-circuit voltage (V_oc_), photocurrent density (J_ph_), and *E*_g_ can be expressed by^49^.


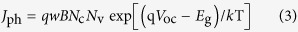


where w is ceramic thickness, B is the radiative recombination factor which is temperature-dependent^50^, q is carrier charge, T is room temperature (300 K), *k* is the Boltzmann constant, and N_c_ and N_v_ are effective densities of states in the conduction and valence bands. These bands can be correlated to intrinsic carrier concentration (*n*_*i*_), i.e.^51^,^52^


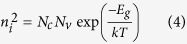


where the reduction of *E*_g_ due to Nd doping in the exponential term is the most important factor for the change in carrier concentration (*n*_i_). N_c_N_v_ may be associated with current leakage and non-radiative recombination^53^,^54^,^55^, and we assume that N_c_ and N_v_ are dopant-independent parameters. (One Nd atom contributes 6 more valence electrons than the Bi atom in BFO.) Fig. S3(a) shows the open-circuit voltage V_oc_ and short-circuit current density (J_sc_) as functions of irradiation intensity from the ITO/BFO/Au and ITO/BFONd/Au heterostructures under irradiation for the same thickness of d = 0.2 mm. V_oc_ and J_sc_ increase rapidly in the low-intensity region, approaching saturation as irradiation intensifies. Since the dark current density (J_dark_) is smaller than the photocurrent density (J_ph_), the approximation of J_sc_  = J_ph_ − J_dark_~J_ph_ can be expected. We used Eq. (3) to estimate the J_ph_ ratio between BFO and BFONd, i.e.


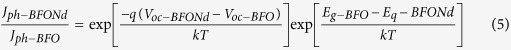


As listed in Table S3, the J_sc−BFONd_/J_sc−BFO_ ratio is positively correlated with irradiation intensity, indicating that the photovoltaic effects in BFONd were enhanced with increasing intensity. This is consistent with the reduction of leakage current in the rare-earth doped BFO^18^. In contrast, the J_ph−BFONd_/J_ph−BFO_ decreases with increasing irradiation intensity. There are two possible mechanisms for the deviation between the J_sc−BFONd_/J_sc−BFO_ and J_ph−BFONd_/J_ph−BFO_ ratios. First, as shown in Eq. (3), the photocurrent J_ph_ is sensitive to leakage current^56^ and intermediate defect states^17^, which are expected to have more profound effects in BFO at lower irradiation intensities, as charged carriers can be trapped in the low-lying defect states below the conduction band^17^. In addition, lower leakage current in BFONd is expected to have higher V_oc_ according to the Schockley^57^ and Poole-Frenkel emission equation^16^. Therefore, the J_ph-BFONd_/J_ph-BFO_ ratio tends to be larger at lower irradiation intensities. Secondly, the non-radiative carrier recombination lifetime (τ_r_) depends on irradiation intensity, decreasing as intensity decreases. The relation between carrier density and recombination time can be expressed by^58^


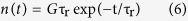


where n(t) is carrier concentration, G is the generation constant, τ_r_ is the carrier recombination lifetime, and t is the time after irradiation. According to Eqs (4) and (6), N_c_N_v_ and *n* are expected to have larger values for longer τ_r_ at lower irradiation intensities, in which the defect states may cause a reduction of photocurrents. At higher irradiation intensities, carrier concentration *n* may reach saturation in both BFO and BFONd, resulting in similar ratios appearing in J_sc−BFONd_/J_sc−BFO_ and J_ph−BFONd_/J_ph−BFO_.

## Conclusions

A-site Nd^3+^ doped BiFeO_3_ (Bi_0.9375_Nd_0.0625_)FeO_3_ (or BFONd) coupled with ITO and Au electrodes has demonstrated a maximal photovoltaic power conversion of ~0.82% while under irradiation of wavelength λ = 405 nm. This is not only the highest in recent PV studies of BFO materials, but is also promising for future application toward photo-driven actuators and optical sensors. The I-V characteristic curve (without irradiation) exhibits a p-n-like behavior and suggests a p-n junction interface between the ITO thin film and BFO (or BFONd) ceramics. The first-principles calculated band gaps for BFO and BFONd are 2.25 eV and 2.23 eV, which are consistent with the experimental band gaps of ~2.24 eV and ~2.20 eV, respectively. The enhanced photovoltaic responses can be mainly attributed to two phenomena: the reduction of the band gap in BFONd and the increase in valence electrons of the Nd atom.

## Additional Information

**How to cite this article:** Peng, Y.-T. *et al*. Remarkably enhanced photovoltaic effects and first-principles calculations in neodymium doped BiFeO_3_. *Sci. Rep.* **7**, 45164; doi: 10.1038/srep45164 (2017).

**Publisher's note:** Springer Nature remains neutral with regard to jurisdictional claims in published maps and institutional affiliations.

## Supplementary Material

Supplementary Materials

## Figures and Tables

**Table 1:** Fractional coordinates and occupancies for the A-site substitution.

A-site case at χ^2^ = 1.625	Fractional coordinates			Occupancy
Bi	0.000000	0.000000	0.003391	0.9375
Fe	0.000000	0.000000	0.226548	1.0000
O	0.444678	0.027363	0.961068	1.0000
Nd	0.000000	0.000000	0.988694	0.0625

**Table 2:** Total free energies of A-site (Bi-site) Nd substitution and interstitial sites (B, C, and D cases).

	A-site	interstitial site B	interstitial site C	interstitial site D
Total free energy (eV)	−505.1281	−504.4567	−504.2697	−504.4567
