# Supplementary material for: Remarkably enhanced photovoltaic effects and first-principles calculations in neodymium doped BiFeO3
Source: Sci Rep. 2017 Mar 24;7:45164. doi: 10.1038/srep45164 (PMC5364538; doi:10.1038/srep45164)
Supplement: Supplementary Materials [file srep45164-s1.pdf]

Supplementary Materials for "Remarkably enhanced photovoltaic effects and first-principles calculations in neodymium doped $BiFeO_3$"

Yi-Ting Peng[1], Shan-Haw Chiou[2], Ching-Hung Hsiao[1], Chuenhou (Hao) Ouyang[1]*, and Chi-Shun Tu[3*]

[1]Department of Materials Science and Engineering, National Tsing Hua University, Hsinchu, Taiwan 300, Republic of China.

[2]Material and Chemical Research Laboratories and Nanotechnology Research Center, Industrial Technology Research Institute, Hsinchu, Taiwan 310, Republic of China.

[3]Department of Physics, Fu Jen Catholic University, New Taipei City, Taiwan 24205, Republic of China.

Table S1 Band gaps for different Hubbard parameters (U).

U (eV)	$E_{g\text{-}cal}$ (eV)	$E_{g\text{-}exp}$ (eV)	Error (%)
5.0	0.350	2.24	84.38
6.0	0.261		88.35
7.0	2.157		3.71
7.1	2.178		2.77
7.2	2.197		1.92
7.3	2.218		1.00
7.4	2.220		0.98
7.5	2.254		0.62
7.6	2.273		1.47
7.7	2.274		1.51
7.8	2.311		3.17
7.9	2.335		4.24
8.0	2.352		5.00
9.0	2.553		13.97

J was obtained based on the reference[42]

Table S2 Fractional coordinates and occupancies for the interstitial substituted B, C, and D cases as shown in Fig. 2.

Interstitial site B	Fractional coordinates			Occupancy
Bi	0.000000	0.000000	0.999528	1.0092
Fe	0.000000	0.000000	0.220732	1.0000
O	0.441192	0.018487	0.951919	1.0000
Nd	0.000000	0.000000	0.694461	-0.092

Interstitial site C	Fractional coordinates			Occupancy
Bi	0.000000	0.000000	0.002864	1.0406
Fe	0.000000	0.000000	0.223014	1.0000
O	0.429957	0.024474	0.961172	1.0000
Nd	0.000000	0.000000	0.837103	-0.0406

Interstitial site D	Fractional coordinates			Occupancy
Bi	0.000000	0.000000	0.000000	1.2015
Fe	0.000000	0.000000	0.220770	1.0000
O	0.442800	0.018700	0.952000	1.0000
Nd	0.625000	0.333333	0.175693	-0.2015

(the negative occupancy indicates impossibility)

Table S3 V_{oc}, J_{sc} , $J_{sc\text{-}BFONd}/J_{sc\text{-}BFO}$, and $J_{ph\text{-}BFONd}/J_{ph\text{-}BFO}$ for various irradiation intensities.

Irradiation intensity (W/m^2)	Compound	V_{oc} (V)	J_{sc} (A/m^2)	$J_{sc\text{-}BFONd}/J_{sc\text{-}BFO}$	$J_{ph\text{-}BFONd}/J_{ph\text{-}BFO}$	Deviation (%)
39	BFO	0.54	0.04	11.57	85.82	74.3
	BFONd	0.62	0.46			
65	BFO	0.56	0.04	15.0	94.90	79.9
	BFONd	0.65	0.67			
140	BFO	0.56	0.05	19.67	89.55	69.9
	BFONd	0.68	1.06			
913	BFO	0.70	0.09	32.81	41.15	8.3
	BFONd	0.76	2.96			
1593	BFO	0.74	0.11	34.79	33.14	1.7
	BFONd	0.79	3.97			

Supplied Materials

Fig. S1 Optical transmission spectra.

Fig. S2 Density of states (DOS) and projection densities of states (PDOS) of (a) BFONd and (b) BFO.

Fig. S3 (a) V_{oc} and (b) J_{sc} as functions of irradiation intensity for BFO (red squares) and BFONd (blue dots).

Fig. S1

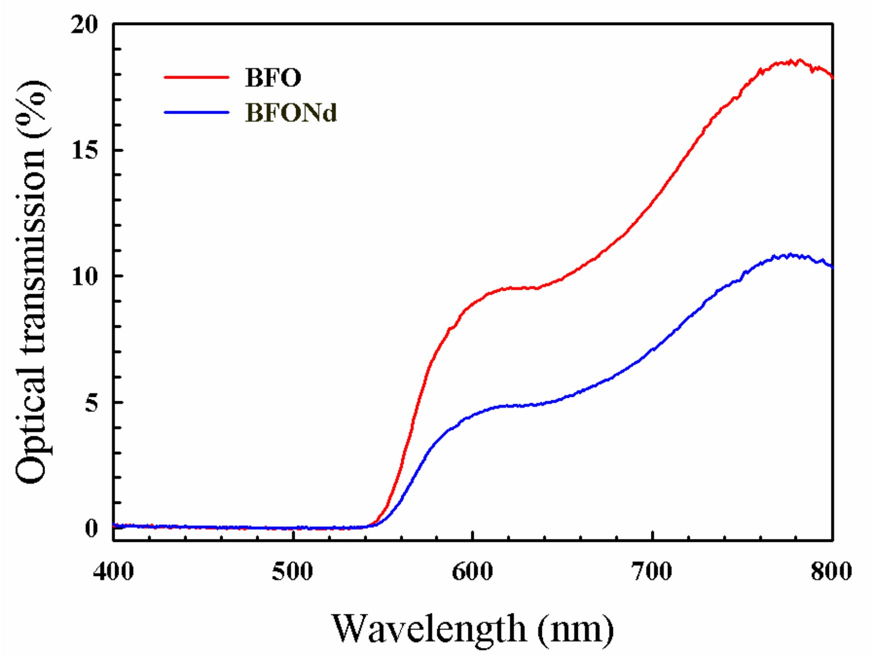

Fig. S2

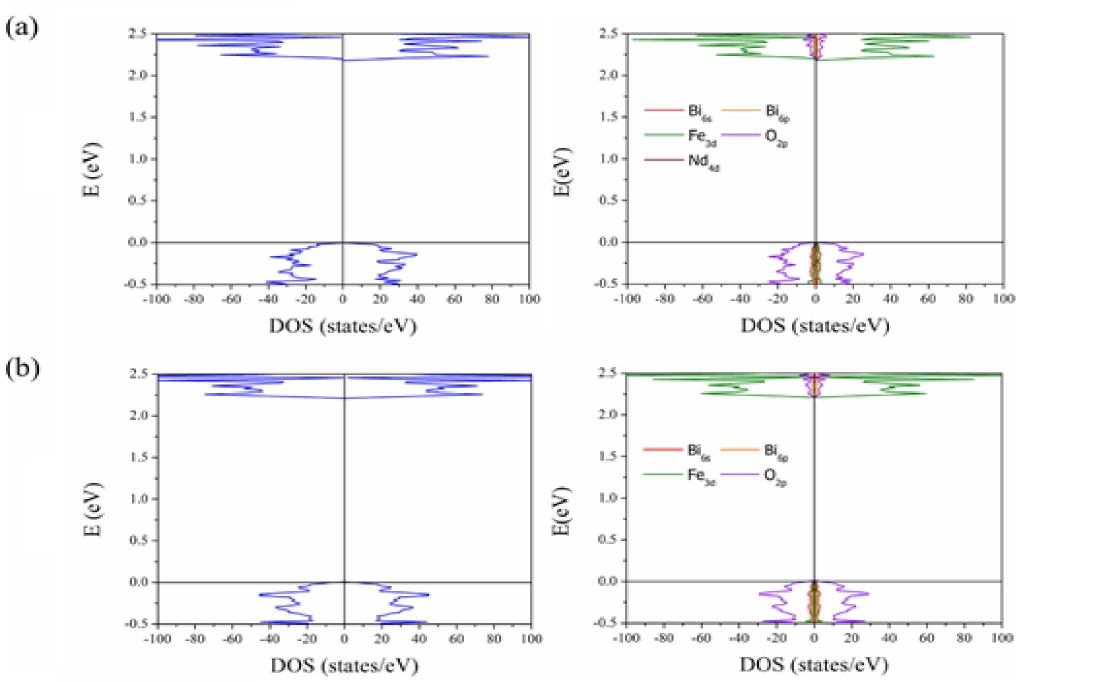
(a)
(b)
E (eV)
E(eV)
DOS (states/eV)
Bi6s
Bi6p
Fe3d
O2p
Nd4d

Fig. S3

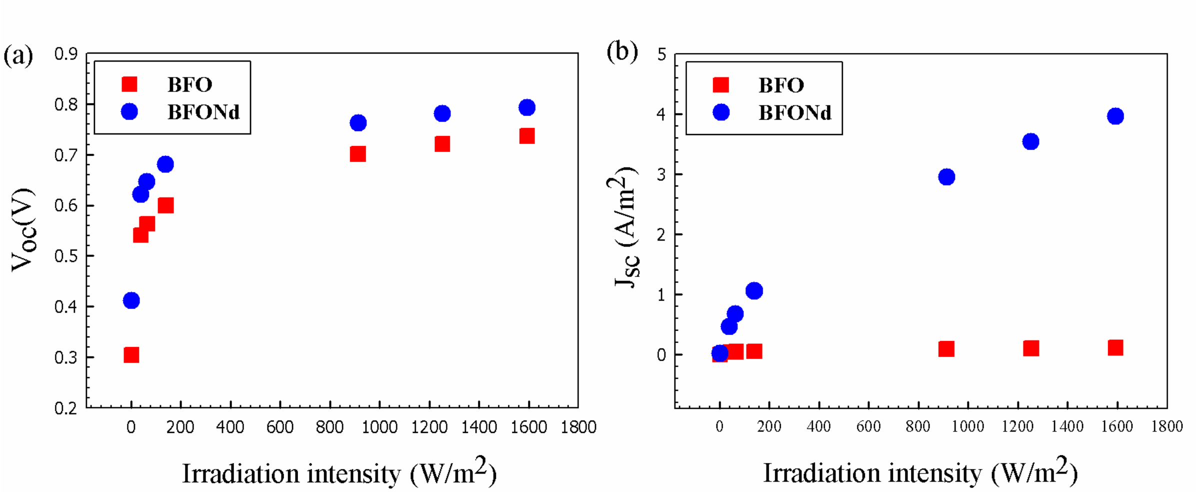
(a)
(b)
BFO
BFONd
Voc(V)
Jsc (A/m2)
Irradiation intensity (W/m2)
